# An Integrative Analysis of the Dynamics of Landscape- and Local-Scale Colonization of Mediterranean Woodlands by *Pinus halepensis*

**DOI:** 10.1371/journal.pone.0090178

**Published:** 2014-02-28

**Authors:** Efrat Sheffer, Charles D. Canham, Jaime Kigel, Avi Perevolotsky

**Affiliations:** 1 Department of Ecology and Evolutionary Biology, Princeton University, Princeton, New Jersey,United States of America; 2 Cary Institute of Ecosystem Studies, Millbrook, New York, United States of America; 3 The Robert H. Smith Institute of Plant Sciences and Genetics in Agriculture, Robert H. Smith Faculty of Agriculture, Food and Environment, The Hebrew University of Jerusalem, Rehovot, Israel; 4 Department of Agronomy and Natural Resources, Agricultural Research Organization, The Volcani Center, Bet Dagan, Israel; University of Tartu, Estonia

## Abstract

Afforestation efforts have resulted in extensive plantations of either native or non-native conifers, which in many regions has led to the spread of those conifers into surrounding natural vegetation. This process of species colonization can trigger profound changes in both community dynamics and ecosystem processes. Our study disentangled the complexity of a process of colonization in a heterogeneous landscape into a simple set of rules. We analyzed the factors that control the colonization of natural woodland ecosystems by *Pinus halepensis* dispersing from plantations in the Mediterranean region of Israel. We developed maximum-likelihood models to explain the densities of *P. halepensis* colonizing natural woodlands. Our models unravel how *P. halepensis* colonization is controlled by factors that determine colonization pressure by dispersing seeds and by factors that control resistance to colonization of the natural ecosystems. Our models show that the combination of different seed arrival processes from local, landscape, and regional scales determine pine establishment potential, but the relative importance of each component varied according to seed source distribution. Habitat resistance, determined by abiotic and biotic conditions, was as important as propagule input in determining the density of pine colonization. Thus, despite the fact that pine propagules disperse throughout the landscape, habitat heterogeneity within the natural ecosystems generates significant variation in the actual densities of colonized pine. Our approach provides quantitative measures of how processes at different spatial scales affect the distribution and densities of colonizing species, and a basis for projection of expected distributions. Variation in colonization rates, due to landscape-scale heterogeneity in both colonization pressure and resistance to colonization, can be expected to produce a diversity of new ecosystems. This work provides a template for understanding species colonization processes, especially in light of anthropogenic impacts, and predicting future transformation of natural ecosystems by species invasion.

## Introduction

The process of species colonization is fundamental in basic ecological questions of successional, metapopulation, and community dynamics (e.g., [1], [2], [3]), as well as in studies of biological invasions [4], conservation [5], restoration (e.g., [6], [7]) and climate change adaptation [8], [9]. The successful colonization of a species in a site can have broad implications for the diversity and abundance of resident species, the structure of the ecosystem, and rates of ecosystem processes [10]–[14]. Furthermore, changes in ecosystem structure and function following colonization by new species can have cascading effects on the distributions of a wider range of species [15], [16]. Species colonization can lead to ecosystem transformation and in some cases to the emergence of a novel ecosystem [13], [17]. The transformation of an ecosystem following its colonization may involve unpredictable thresholds, feedbacks and state transition [18], thus stressing the importance of understanding how the rates of colonization affect the abundance of colonists which in turn determines their potential engineering effects and transforming impacts (e.g., [19], [20]).

Colonization can be viewed as the net result of processes starting from propagule production and ending in the survival to reproductive maturity of the colonist [21], [22]. The factors that control plant recruitment can be structured in terms of the large-scale factors that determine propagule pressure – i.e. the rate of propagule arrival (propagule number, *sensu* [23]) [24],[25], and local factors that determine the resistance of the host community to the establishment and survival of colonists [26]. This is a useful simplification when there is limited information on all intermediate stages of the colonization process (e.g., seed dispersal, germination and establishment), or for highly variable systems (e.g., spatial heterogeneity).

The study of the factors that control colonization inherently requires a landscape perspective, first, to account for possible sources of propagule pressure, and second, to explain heterogeneous patterns of colonization. We present an approach to discern how processes at different spatial scales determine the patterns of species colonization in heterogeneous landscapes. Our approach is based on (A) quantifying successful establishment of the colonizing species, and (B) identifying and quantifying the range of factors that control species recruitment across spatial scales [7]. We emphasize the importance of understanding how the combined effect of factors ranging from landscape-scale propagule pressure (e.g., [27]) to local resistance act in concert to determine colonization densities across the landscape. The outcomes can thus be used to compose abundance-maps of the colonists [28], and to study changes in species distributions following human impacts on climate and landscapes.

Here we present an empirical data-based model of species colonization, focusing on colonization by plant species, but the same approach can be applied to other taxa. As a part of a broader analysis of the spatial dynamics of human-altered landscapes [7], we focus here on the process of colonization of natural ecosystems by tree species from planted forests, as a first and critical stage of ecosystem transformation. We studied these dynamics in a Mediterranean landscape, a case study that represents a highly heterogeneous spatial mosaic of planted forests and natural Mediterranean woodlands (maquis [29]) as a result of human impacts [30]. We applied an inverse modeling approach to fit a nested set of statistical models of the processes controlling the densities of colonists of a planted pine species in natural woodlands. Specifically, we address two broad questions: (1) how does the landscape configuration of propagule sources (spatial distribution, abundance, and attributes of individual seed source areas), at various spatial scales, influence the potential colonization of natural woodlands? and (2) how do local biotic and abiotic conditions of the natural woodlands control (resist or promote) colonization by these pines? Comparison of alternative models allowed us to test hypotheses on the importance of different processes in determining successful colonization. We searched for the most parsimonious model that provides a quantitative and ecologically significant explanation of these controlling factors. Finally, we show how the results can be used to generate species abundance maps and to predict future composition and structure of the colonized ecosystems.

## Methods

### The study system

Over the past century there have been two major patterns of land-use change in the northern Mediterranean Basin: (*i*) a decrease in the intensity of traditional land-use (grazing, wood-cutting and agriculture), in some cases combined with management (e.g. fencing, preservation) to allow recovery of native communities; and (*ii*) extensive planting of forests on degraded sites [31], [32]. As a result of these changes, the vegetation of the Mediterranean landscape of Israel is currently composed of a spatial mosaic dominated by two very different ecosystems: evergreen shrublands and woodlands with a diversity of sclerophyllous Mediterranean trees and shrubs (mostly dominated by oaks) interspersed with patches of herbaceous, mostly annual vegetation, and plantations of conifers, primarily the native *Pinus halepensis* Mill. and *P. brutia* Ten. Planted forests now cover approximately 12% of the Mediterranean landscape in Israel, while sclerophyllous woodlands and shrublands cover almost 20% of the area [33].

**Figure 1:**
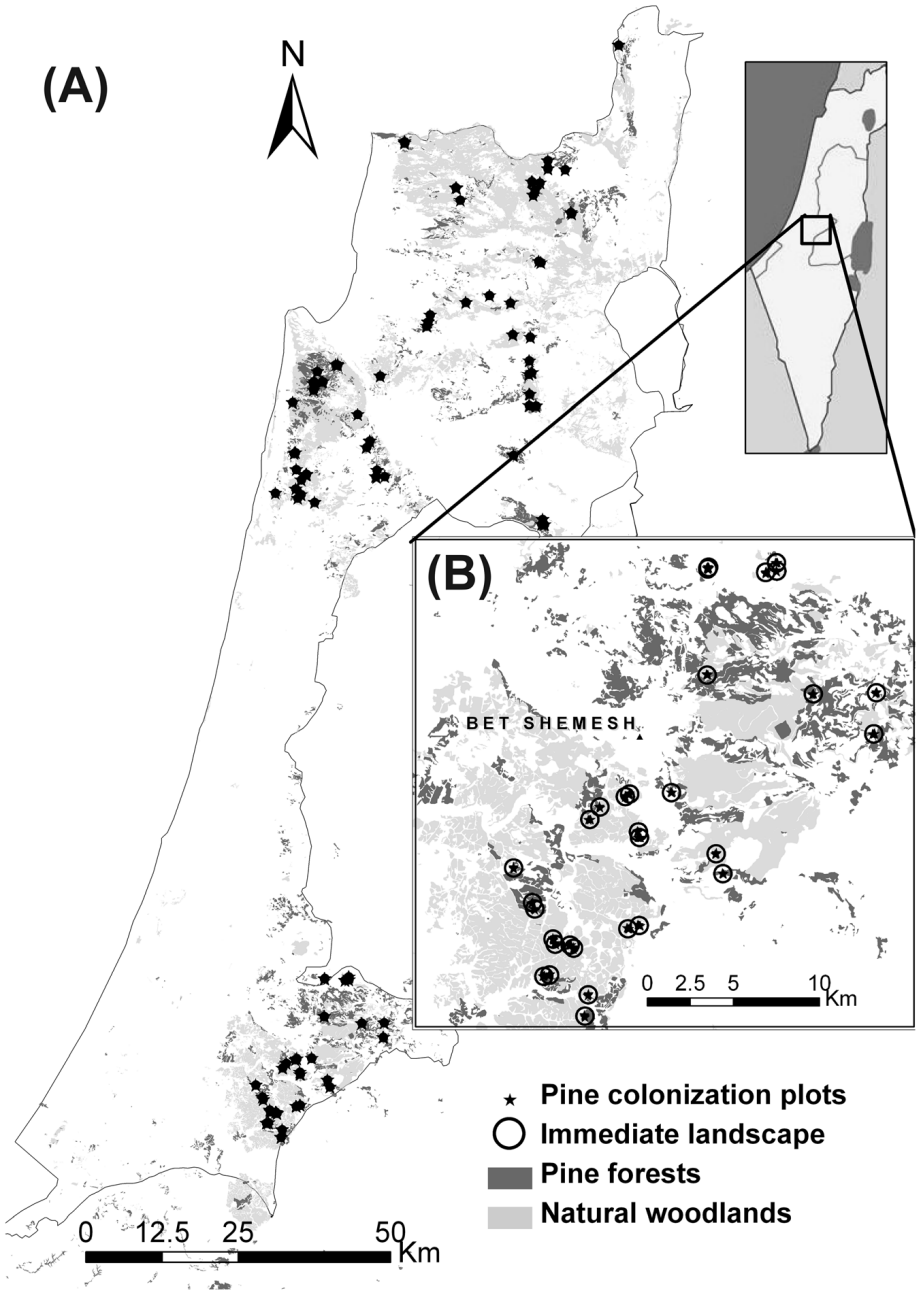
Map of the distribution of Mediterranean sclerophyllous woodlands and shrublands and planted pine forest in the study area. The distribution of forests and woodlands and all sampled sites (stars) is shown on the entire map of the Mediterranean region of Israel (A), with a zoom into the area of the Judean Mountains (B). The immediate landscape buffers (black circles) mark a 500 m radius area surrounding each plot. Other land covers (e.g., infrastructure, agriculture, meadows, urban) are not shown (white areas in the figure).

The juxtaposition of woodlands and pine forests within the Israeli landscape (Fig. 1) has created opportunities for reciprocal colonization of each ecosystem type by dominant species of the other community [7], [34], [35], and hence provide a good case study for the process of species colonization. Since both natural ecosystems and planted forests in our studied landscape are the product of recent processes (that occurred in the last century), their history is well documented and detailed spatial information is available.

### Field survey

We conducted a detailed field survey (2008–2009) to measure the spatial distribution and densities of colonizing *P. halepensis* individuals in 470 plots (8 m radius, 200 m^2^) distributed in 94 sites (authorized by the Israeli Nature and Parks authority for reserves and parks, some locations did not need any permit). Studied sites included a variety of natural shrublands and woodlands throughout the Mediterranean region of Israel (*ca*. 710,000 ha, Fig. 1), but avoiding pine stands and areas in which disturbance (mainly fire or human action, in the last 10 years) might have affected pine colonization. In each site, five plots were distributed along a single transect, with >50 m distance between plots. We stratified the sampling effort for the full range of factors included in our models: (1) a precipitation gradient (400–900 mm per year); (2) the proximity to pine stands (0–2300 m distance to nearest pine seed source); (3) the type of vegetation and woody vegetation cover; (4) rock-soil formation (calcareous Red Brown Mediterranean “Terra-Rosa” Soil [USDA Rhodoxeralf or Haploxeroll, FAO luvisol] formed on limestone or dolomite bedrocks, vs. chalk and marl based Rendzina soils [USDA Haploxeroll or Xerothent]); and (5) presence/absence of grazing by cattle, goats or sheep.

### Sampling protocol

In each plot we measured the densities of *P. halepensis* colonists and the abiotic and biotic conditions. We recorded GPS coordinates, rock and soil type, and evidence of current grazing by cattle, goats or sheep (fencing, signs of plant browsing, animal excrement and soil trampling). We also measured (a) the cover of all woody vegetation along one 16 m transect crossing the plot in a random direction, to estimate the percentage of woody cover; and (b) the following characteristics of all pine trees >50 cm high (representing established individuals): tree height, diameter at breast height (for trees ≥1.3 m high), trunk base diameter, reproductive stage, number of branch whorls, length increment in the two last growth seasons, and type of micro-habitat in which the tree was growing (soil, rock, woody plants). For the analyses presented here we used the number of pine colonists per plot, the number of branch whorls as a surrogate for tree age (in units of years), and the diameter at breast height combined with tree reproductive state to calculate the total basal area of reproductive pine colonists within each plot (in units of m^2^).

### Data preparation and analysis

#### Environmental data

The data for each plot was complemented with environmental attributes from mapped data sources of bedrock and soil type (Israeli GIS surveys) and mean annual precipitation estimates (in units of mm year^−1^) using an interpolation model [36].

#### Map of P. halepensis seed source areas

We assembled a map of the configuration of all pine seed sources in the study region. The map is a compilation of maps of all planted forests of the Israeli Forest Service (KKL), natural vegetation associations (Israel Nature and Parks authority), ancient pine stands [37], and any additionally known *P. halepensis* stands which were not mapped in any of the previous sources, e.g. pines in parks, settlements and urban areas. To map these additional pine patches we compared all areas mapped as covered by trees in a land-use map (Ministry of Agriculture of Israel) with the maps of planted forest and natural vegetation. To find any additional *P. halepensis* seed source areas we carried out a meticulous examination of all the polygons of tree cover in the land-use map within 5 km distance from each of the survey plots and added polygons occupied by *P. halepensis*. For all *P. halepensis* seed sources we determined average tree age (according to the year of plantation) and estimated the proportion of canopy trees represented by *P. halepensis*. We converted the unified map into 20×20 m cell size raster grid maps (one for pine age and one for proportion of pines). All the above procedures were done in ArcMap 9.2 and ArcInfo 9.3 [38].

### Maximum likelihood analyses of pine colonization

We analyze if and how the number of *P. halepensis* colonists at each plot is affected by: (I) the GPS location of the plot and thus the configuration of pine seed sources in the landscape that surround it; (II) the basal area of reproductive pine colonists within each plot; (III) the effects of the abiotic conditions of soil or rock type and precipitation; and (IV) biotic conditions of grazing type and intensity and cover of woody vegetation. We used maximum-likelihood methods to predict the number of *P. halepensis* colonists in each plot as a function of how colonization dynamics are controlled by (1) propagule pressure, and (2) local environmental conditions acting as resistance factors, as used in a parallel study of oak colonization in pine forests [7]. This inverse modeling approach is a form of statistical modeling that searches for the best scientific model and the maximum-likelihood estimated set of parameters for that model given a large empirical dataset.

#### Propagule pressure

We tested how pine propagule pressure (*P*) is determined by the amount of seed input into the colonized plot from three propagule sources: a constant regional input (*P_reg_*), a landscape input (*P_lan_*) determined by the spatial configuration of seed sources in the landscape and the seed dispersal kernel, and a local input from reproductive trees within the sampled plot (*P_loc_*) (equation 1):
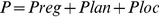
(eq.1)


We conducted a preliminary analysis to identify the optimal extent for the analysis of landscape propagule pressure. We used different ranges (from 500 to 5000 m) for the radius of the area surrounding each focal plot for which the input of propagules from all seed sources in the surrounding landscape was calculated. This analysis showed that the spatial effects of *P. halepensis* seed sources more than 500 m away from the plot did not contribute to the overall likelihood of a model, which coincides with previous studies showing that the majority of pine seed dispersal falls within 100 m from the parent tree [39], [40]. Thus, the components of colonization pressure can be defined as: a constant for *P_reg_* (distance > 500 m.), a distance dependent *P_lan_* (< 500 m) and depending on the basal area of reproductive trees within the plot (*P_loc_*). The landscape propagule pressure to a plot was modeled as the sum of spatially explicit inputs from all pine seed source grid cells in the 500 m radius area around each plot. For each pine source grid cell we tested the effect of three attributes: (1) distance to the colonized focal plot, (2) stand age, and (3) proportion of pines in the stand. Initial tests indicated that the most important effect for landscape propagule pressure was distance to the plot (model 4 vs. others in Table 1).

**Table 1:** Model comparison.

	Num. of		Mean	*Propagule pressure (P) sources*			*Potential colonization factors*			
Model	Parameters	AICc	*R^2^* *	*Regional* †	*Landscape* ^‡^	*Local*	*Precipi tation*	*Rock-Soil* §	*Grazing*	*Woody cover* ¶
1.	17	1492.32	0.18	<100	Exp-Distance	+	+	4 rock	2	LN-Threshold
2.	15	1493.07	0.16	<100	Exp-Distance	+	+	2 soil	2	LN-Threshold
3.	17	1496.84	0.18	<1000	Exp-Distance	+	+	2 soil	4	LN-Threshold
4.	19	1501.19	0.2	<1000	Exp-Distance + Age	+	+	2 soil	4	LN-Threshold
5.	16	1501.61	0.18	<1000	Exp. Distance	+	+	2 soil	4	Exponential
6.	18	1503.14	0.12	<1000	Anistropic-LN-Distance	+	+	2 soil	4	Exponential
7.	16	1503.67	0.19	<1000	LN-Distance	+	+	2 soil	4	Exponential
8.	23	1504.31	0.15	<100	Anistropic -Exp-Distance	+	+	2 soil	2	LN-Threshold
9.	16	1569.95	0.04	<1000	S.I.-Total-Pine	-	+	3 soil	4	LN-Threshold
10.	15	1576.32	0.03	<1000	S.I.-Total-Pine	-	+	2 soil	4	LN-Threshold
11.	12	1582.58	0.03	<1000	S.I.-Total-Pine	-	+	2 soil	4	-
12.	12	1673.83	0	<1000	S.I.-Total-Pine	-	-	2 soil	4	Exponential

The best models (lowest AICc) are indicated in boldface type. A ‘+’ or ‘-’ sign indicates the inclusion or exclusion of that factor in the model, respectively. The number of categories included in each model for the analyzed factor is listed under rock-soil and grazing effects and the functional form used is listed for all other effects.

*Mean *R^2^* – average of 10,000 *R^2^* calculations of a subset of the dataset that includes all results with pines and a randomly drawn subset of the results with zero pine colonization as determined by the zero-inflated distribution of the data (1 – *pz*).

† Regional propagule pressure (***P_reg_***) bounded to be <100 or bounded to <1000.

Landscape propagule pressure (***P_lan_***) modeled using either spatially explicit distance-dependent models: an exponential (“Exp-Distance”) Weibull kernel, with or without the effect of the age of pines in the seed source (“+ age”), an isotropic or an anisotropic lognormal (“LN-Distance”) kernel, or an anisotropic exponential kernel skewed in 8 wind directions (“Anistropic-Exp-Distance”); or a spatially implicit (“S.I.”) distance independent model in which regional pressure is a linear function of total pine cover in 500 m distance from sample.

§ Number of rock or soil categories. Soil categories include Terra-Rosa and Rendzina (2 categories) or Terra-Rosa, light Rendzina and Brown Rendzina (3 categories). Rock categories include Chalk, Marl, Dolomite and Limestone.

¶ Resistance by woody cover modeled as an exponential or a lognormal (“LN-threshold”) with a lower threshold for which *f (V<Vthreshold)*  =  0.

#### Alternative dispersal functions

We tested two functions, exponential and lognormal, for the landscape-scale dispersal curves [33], [40], [41]. The exponential function consistently had higher likelihood than the lognormal function (ΔAIC_c_ = 6.83 for the exponential model, models 3 and 7 in Table 1), and was selected as the basis for further model development. The exponential dispersal function was modeled as: 
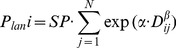
(eq.2)where the landscape input to the *i* ^th^ plot (*P_lan i_*) is a sum of inputs from all pine source cells *j* = 1…*N* within 500 m of the plot location, *SP* is a parameter that represents the average input from a pine seed source immediately adjacent to the sample (at *D_ij_* = 0), *D_ij_* is the distance of the *j* ^th^ pine source cell from plot *i*, and *α* and *β* are parameters determining the shape of the function.

For a wind dispersed species such as *P. halepensis*, the landscape component of propagule pressure may be affected by directional winds during seed release [24], [41]. We examined two additional models to test the potential importance of anisotropic wind direction effects as an alternative to the simple isotropic dispersal function described above: an anisotropic log-normal model skewed towards a single main wind-dispersing direction [12]; and a negative exponential model asymmetrically skewed in eight cardinal wind directions according to eight linear slope parameters (equation 3) (Table 1 models 6 & 8 respectively).
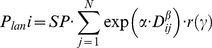
(eq.3)where *r*(*γ*) is a vector of eight parameters ranging 0–1, starting at due north and going clockwise in the wind-rose surrounding each seed source cell, and *γ* is the angle from the pine seed source area to the *i* ^th^ plot. A value of *r = *1 indicates maximum potential seed input and zero indicates no seed input to that direction.

The contribution of seeds from stands at distances greater than 500 m is implicitly incorporated in the regional propagule pressure (*P_reg_*), which is constant throughout the study area. We compared models with different upper limits for the regional propagule pressure (100–10000 propagules per plot), to test for the importance of restricting the general propagule pressure from overwhelming total *P* (models 2 & 3 in Table 1).

For the local propagule pressure (*P_loc_*) we used a simple linear model in which propagule pressure to the *i* ^th^ plot varied as a function of the basal area of locally established reproductive pines in plot *i*, with slope given by the parameter *λ*.

#### Resistance to colonization

We tested the impact of local conditions acting as resistance factors that reduce potential colonization relative to propagule pressure (*P*). We used multiplicative models for the effects of biotic (grazing, woody vegetation) and abiotic conditions (rock-soil type and annual precipitation) considered to be important in Mediterranean ecosystems (equation 4):
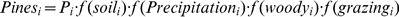
(eq.4)where *Pines_i_* is the predicted number of pine colonists, and *P_i_* is the total colonization pressure in plot *i.* The filtering effect imposed by each factor ranges from 0–1 and scales propagule pressure so that high values imply facilitation (high potential colonization) and small values represent strong resistance to establishment. To understand the relative contribution of each of these local conditions to colonization resistance we searched for the most parsimonious grouping of the four effects and different partial combinations of them (e.g., Table 1). The use of a multiplicative model for the effects of all variables allows testing for the independent effect of each variable as well as all possible interactions among variables. However, if there is strong covariation between variables (e.g. soil type and vegetation cover) then model comparison would show that one of these variables is redundant and a simple model with just one of the two collinear variables is more parsimonious.

The effects of rock-soil type and grazing regime were included as categorical parameters. We used four grazing categories: no grazing, light cattle or sheep grazing, moderate cattle grazing only, and intensive cattle or goat grazing. After finding very similar parameters for the first and the last two categories we tested a simpler model in which grazing regimes were lumped into two categories: none to low cattle or sheep grazing vs. medium to intensive cattle or goat grazing (models 2 & 3 in Table 1). We used a Gaussian model for the effect of precipitation on pine colonization (equation 5), based on the observation that *P. halepensis* is drought tolerant [42], [43] which can imply low sensitivity to precipitation above a certain threshold.
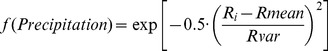
(eq.5)


This form provides a very flexible function, where *R_i_* is mean annual precipitation in the *i* ^th^ plot, *Rmean* is a parameter that determines the precipitation at which maximum potential colonization occurs (lowest resistance), and *Rvar* is a parameter that determines the width of the function.

We tested five competing models for the effect of woody vegetation cover (*V*) on colonization resistance: linear, logistic, Gaussian, exponential and lognormal. The four last models showed an abrupt transition from strong resistance in plots with low woody cover to no resistance in plots with higher woody cover. Based on these results we tested another model with a threshold (*V_th_*) for which *f*(*V_i_* ≤ *V_th_*) = 0 (complete resistance) and a lognormal function for areas in which vegetation cover is above the threshold:
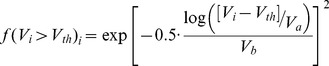
(eq.6)where *V_i_* is the percentage cover of woody plants in the *i* ^th^ plot, *V_a_* is a parameter that determines the percent of woody vegetation cover above the threshold at which maximum pine colonization occurs, and *V_b_* is a parameter that determines the spread of the potential colonization around this maxima. We also tested for the effect of different components of the woody vegetation (e.g. only tree or only shrub covers) as possible surrogates for light availability.

### Parameter estimation and model comparison

We solved for the maximum likelihood estimated (MLE) parameter values using simulated annealing in the likelihood 1.3 package in R [44]. The error terms (*ε*) for the colonization data were modeled using a zero-inflated Poisson distribution [45] after excluding any covariates that could explain the events of zero pine colonization. We compared alternative models (part of which are shown in Table 1) on the basis of their Akaike information criterion corrected for a small sample size (AIC_c_) [46]. We used asymptotic 2-unit support intervals to assess the strength of evidence for individual maximum likelihood parameter estimates [47]. To evaluate model goodness of fit we calculated the R^2^ of the regression of observed vs. predicted for randomly chosen subsets of the data omitting a zero-inflated proportion of the samples (*pz* in Table 2) with zero colonization. We repeated the calculation of R^2^ 10,000 times, each time with a different randomly drawn subset of the dataset and calculated mean R^2^ of all iterations. All analyses were done using the R programming environment version 2.8.0 [48].

**Table 2:** Set of maximum likelihood estimated (MLE) parameters and parameter support intervals for the most parsimonious models.

Parameter	Meaning	MLE (Lower – Upper S.I.)
*α*	Weibull function scale parameter	0.0242 (0.022 – 0.026)
*β*	Weibull shape parameter	1.005 (1 – 1.015)
*SP*	Propagule pressure from a 20×20 m pine source cell at distance = 0	14.289 (11.574 – 16.346)
*Preg*	Regional propagule pressure constant	51.095 (45.475 – 60.409)
*λ*	Linear slope of local propagule pressure [colonists per 1 m^2^ basal area of reproductive trees]	417.2 (183.5 – 735.9)
*V_th_*	Threshold of woody vegetation cover with complete resistance [% cover]	15.774 (14.384 – 16.110)
*V_a_*	Woody cover above *V_th_* of maximum potential colonization [% cover]	0.000 (0.000 – 0.000)
*V_b_*	Standard deviation of lognormal woody effect > *V_th_* [% cover]	13.845 (13.015 – 14.818)
*Rmean*	Mean of Gaussian precipitation effect [mm year^−1^]	739.281 (724.495 – 754.067)
*Rvar*	Variance of Gaussian precipitation effect [mm year^−1^]	161.476 (150.172 – 179.476)
*r1*	Resistance of Chalk substrate	0.497 (0.437 – 0.547)
*r2*	Resistance of Dolomite substrate	0.213 (0.181 – 0.280)
*r3*	Resistance of Limestone substrate	0.451 (0.383 – 0.528)
*r4*	Resistance of Marl rock substrate	0.960 (0.518 – 1)
*s1*	Resistance of Terra-Rosa soil	0.504 (0.433 – 0.558)
*s2*	Resistance of Rendzina soil	0.841 (0.748 – 0.925)
*g1*	Resistance of no grazing or low sheep or cattle grazing	0.136 (0.120 – 0.157)
*g2*	Resistance of moderate and intensive cattle or goat grazing	0.255 (0.229 – 0.282)
*pz*	Increased probability of zero colonization	0.457 (0.393 – 0.511)

For the resistance factors, a low value indicates strong resistance, i.e. low colonization, and high values (resistance→1) correspond to low resistance, i.e. high colonization potential. The list includes all the parameters for the best model (model 1 in Table 1), and the parameters for the effect of soil from the second best model (model 2 in Table 1).

Parameters *r* and *s* are for either model 1 or model 2 (Table 1), respectively.

### Generating abundance maps

We used the most parsimonious model (model 2 Table 1 with MLE parameters from Table 2) to generate a map of the expected densities of *P. halepensis* colonists in the entire Mediterranean region of Israel. We generated three different maps. First, we computed a map of the propagule pressure of *P. halepensis* by calculating the expected propagule pressure at each 20×20 m cell as a sum of the constant regional propagule pressure and the distance-dependent landscape propagule pressure. We calculated the later as the sum of propagule inputs contributed by each pine seed source cell surrounding the focal cell to a distance 500 m (based on the seed source maps described above and the parameters of the best model). We did not calculate local propagule pressure since we have no data on the distribution of mature *P. halepensis* tree colonists outside forests in the studied landscape. To generate a second map of the expected resistance to pine colonization, we created a polygon layer that intersected maps of soil type, precipitation, vegetation type and grazing in the entire region (14,857 polygons). We classified each of these maps to categories that match our model categories and calculated the model predicted resistance to colonization in each polygon (omitting areas that are outside the scope of our analysis, mainly other soil types not included in the model). Third, we used the first two maps to calculate the expected abundance of *P. halepensis* in each grid cell in the Mediterranean region of Israel.

## Results

*Pinus halepensis* colonists >50 cm tall occurred in 40% of the plots sampled. Successful colonists were more common at the site scale (i.e. we found at least one successful colonist per site in 73% of the sites). The maximum likelihood estimate of the probability of absence of successful colonists, regardless of the local abundance of pine seed sources was 0.457 (*pz*, Table 2). Densities of these pines, when present, were 170±225 mean±SD individuals per ha (ranging from 50–1800, 95% C.I.: 135–200 individuals per ha). The age structure of the pines shows a peak in the 15–20 year age classes (median 15, 18.1±12.0 mean±SD, range 2–64) (Fig. S1) suggesting a temporal pattern whereby the rate of colonization changed through time. The most parsimonious model included propagule pressures from all three spatial scales and the four resistance factors (slope of observed to predicted  = 1, mean R^2^ = 0.18 and 0.16 for models 1 and 2, respectively, Table 1). Goodness of fit of this model is high considering the large spatial extent of our analysis and the variability it encompassed.

### Impacts of landscape structure on propagule pressure

**Figure 2:**
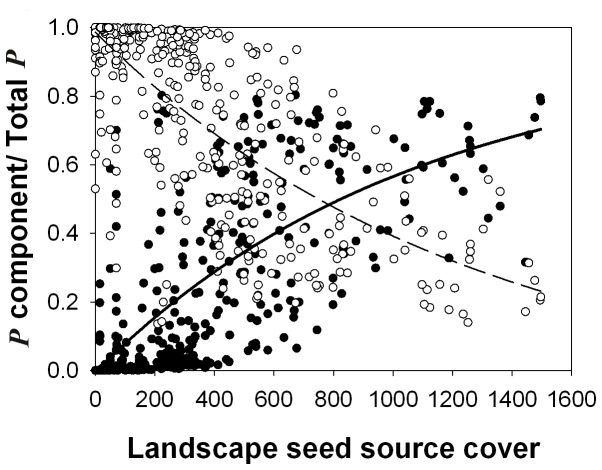
Sources of *Pinus halepensis* propagule pressure. The proportion of regional and landscape components of the propagule pressure from the total propagule pressure as calculated by the most parsimonious model for all 470 sampled plots, as a function of the density of pine seed sources in the 500(m^2^ cover). Proportion of the regional propagule pressure is shown in open circles and a dashed (declining) line, and landscape propagule pressure is shown in filled circles and a black solid (increasing) line (*n* = 470). Best fitted lines are exponential 3 parameter functions (p<0.0001).

Our models show that all three components of propagule pressure are important for the prediction of pine colonization: (*i*) the regional input (*P_reg_*) that is independent of the landscape abundance of seed sources, (*ii*) the landscape input (*P_lan_*) that varies according to the configuration of pine seed sources within 500 m; and (*iii*) the local input (*P_loc_*) from mature pine colonists in sample plots. But, as expected, the relative importance of the three components varied depending on the landscape-scale cover of pine seed sources (Fig. 2). The relative contribution of the landscape propagule pressure compared to the total or the regional input increased in areas with abundant nearby pine seed sources, while the importance of the relative regional input decreased (Fig. 2).

**Figure 3:**
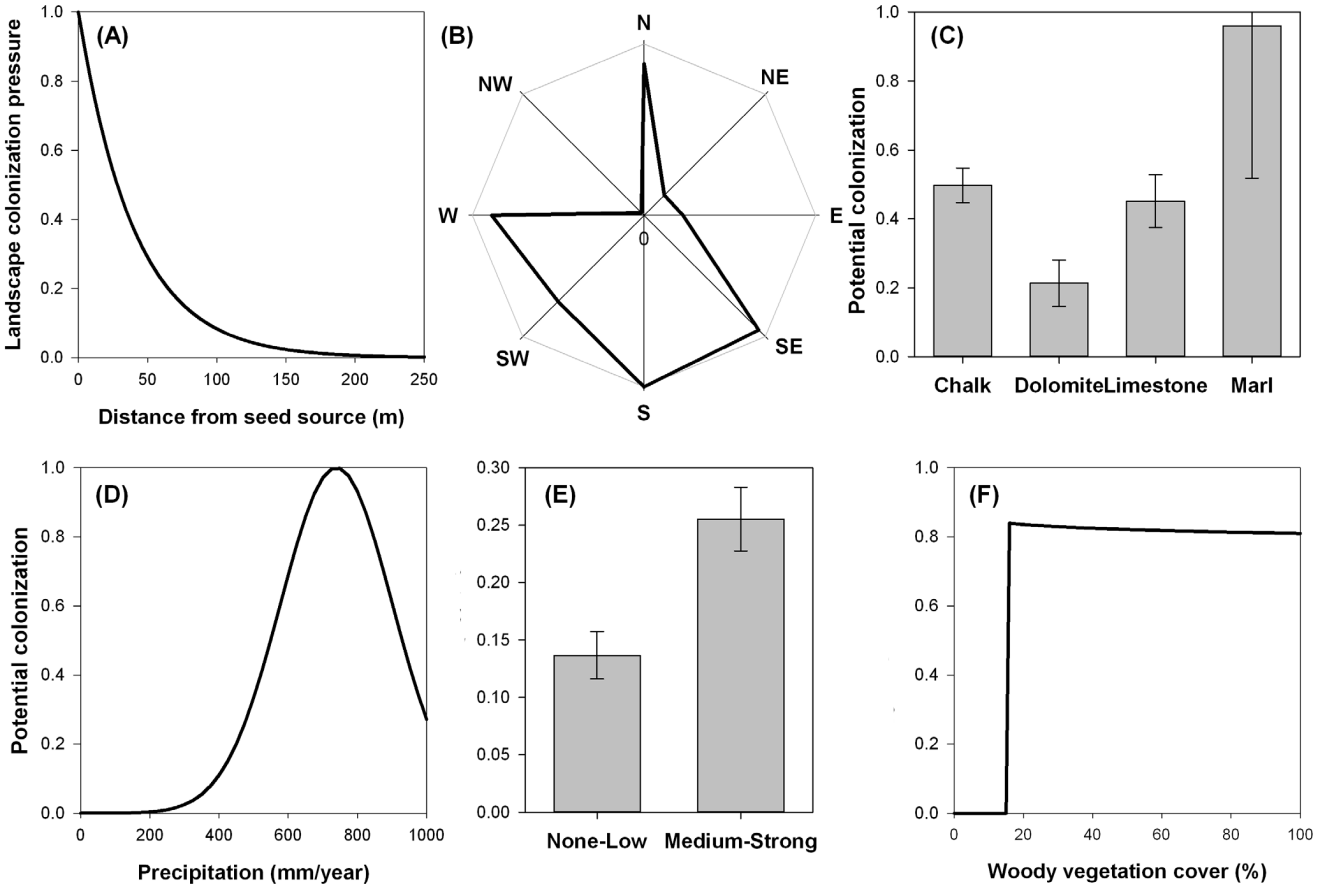
Predicted functional forms of the components of the most parsimonious pine colonization model. (A) Exponential distance dependent decay of landscape propagule input (proportion of seed input per plot relative to maximum input from a seed source stand at distance  =  0) as a function of distance from the pine seed source. (B) Predominant seed dispersal directions. Potential colonization as a function of the effects of local resistance factors including: (C) Bedrock type (parameter ± 2-unit support intervals), (D) Gaussian effect of mean annual precipitation, (E) Grazing regime (parameter ± 2-unit support intervals), and (F) Mediterranean woody vegetation cover. The potential colonization as a function of resistance factors is the relative effect by which each factor scales (decreases) the propagule pressure (ranging from 0–1).

Propagule pressure from landscape seed sources declined exponentially with distance (Fig. 3A), dropping to near zero by 200 m. Models that incorporated stand ages and density of the pine seed sources did not improve the predictions of colonization success (ΔAIC = 3.4, Table 1). For sites >200 m from the nearest pine seed source, the background regional input (via long-distance seed dispersal) constituted the primary source of propagule pressure, indicating that low densities of pine colonization can potentially occur anywhere in these landscapes. Our data do not provide evidence for a strong anisotropic effect of wind direction on dispersal. The results of the more flexible exponential model with eight wind directions indicate that pine seeds disperse preferentially to the west, south and south-east (Fig. 3B). The simpler lognormal model skewed in one direction estimated a single prevailing dispersal direction that was intermediate between the two directions estimated by the more flexible model. However, both anisotropic models were inferior in terms of AICc to the simpler isotropic model (models 6 & 2, Table 1), indicating that wind direction effects played only a minor role at best in the observed distribution of colonists at the large scale of our analysis.

We found only a minor contribution to the overall propagule pressure from reproductive pines that had already successfully established within the natural woodlands (averaging <2% of the overall propagule pressure, ranging 0–41% among individual plots). The estimated magnitude of input from these local seed sources increased linearly with total basal area of mature pines within the sample, although the support intervals for the maximum likelihood estimates of this effect were quite large (parameter *λ* in Table 2).

### Impacts of local habitat resistance on colonization potential

Biotic and abiotic conditions in the woodlands control the potential colonization by either resisting or facilitating colonization by pines. Our models show that the effects of all four sets of factors tested are significant in controlling the density of colonists (Table 1). These factors were not redundant, despite of some collinearity between precipitation and woody vegetation cover, and between precipitation and soil type (Table S1). The two most parsimonious models were similar in most of their components, differing only in the details for the resistance by either rock (four categories, model 1) or soil (two categories, model 2) substrates. Specifically, for a given propagule pressure, potential colonization was twice as high in sites with Rendzina soils (parameter *s_2_* = 0.84) *versus* the red-brown calcareous “Terra-rosa” soil (parameter *s_1_* = 0.5, Table 2). Colonization potential was high in sites with marl bedrock, while sites with chalk and limestone bedrock reduce the potential for colonization, and Dolomitic sites had the highest resistance to pine colonization (Fig. 3C). Our model predicts the most favorable conditions for colonization at intermediate levels of precipitation (∼700 mm annual precipitation, *Rmean* Table 2; ΔAICc = 91.24 for a model with and without the effect of precipitation). Potential colonization decreased at both lower and higher precipitation (Fig. 3D).

Disturbance is typically assumed to alter habitats in ways that create conditions which facilitate colonization of pioneer species or species invasion, either by increasing resource availability or indirectly by reducing various forms of recruitment limitation [49], [50]. Our results indicate that livestock grazing, i.e. intensive cattle and goat grazing, did indeed increase the potential for pine colonization (Fig. 3E). We found strong resistance to pine colonization in habitats without grazing, or with low grazing impacts (e.g. sheep or sparse cattle grazing).

There was a threshold in the effect of woody vegetation cover on colonization. Pines do not appear to be able to colonize sites with low woody cover (<16%, Fig. 3F, Table 2; ΔAICc = 6.26 for a model with and without the effect of woody vegetation cover, and ΔAICc = 6.12 for a lognormal model with and without a threshold). Potential colonization increases sharply above 16% cover, and then decreases very gradually as woody cover increased. We found very low support for alternative models that included only the cover of trees or the structure of the woody vegetation (e.g. woodland vs. shrubland), as possible surrogates for light availability (not shown).

#### Maps projecting P. halepensis colonization

**Figure 4:**
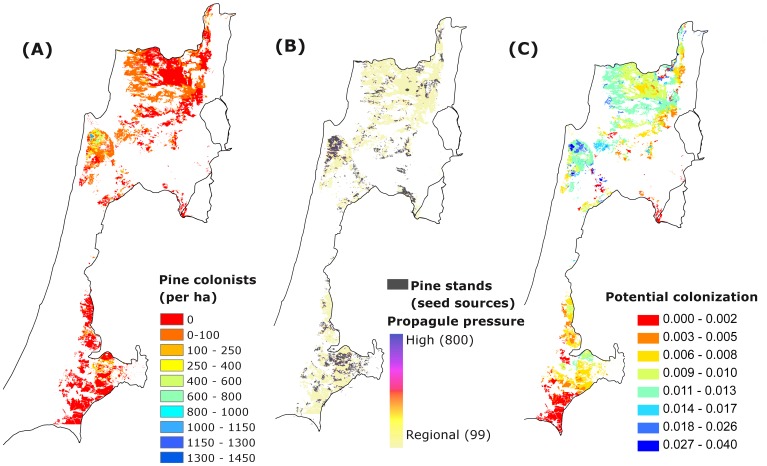
Maps of expected *Pinus halepensis* colonization. Map of the expected distribution of densities of pine colonists (trees ha^−1^) (A), calculated for each location in the Mediterranean region of Israel based on the predictions of the most parsimonious model for: (B) propagule pressure (number of propagules per 200 m^2^ plot) – as a function of the regional propagule input and the distance-dependent input from pine seed sources in the landscape; and (C) potential colonization – calculated by the combined effects of local habitat factors (soil type, precipitation, grazing and woody vegetation cover). White areas in the map are outside the scope of the analysis (developed or agricultural land or different soil type).

The map of the expected densities of *P. halepensis* colonists in the entire Mediterranean region of Israel shows high heterogeneity of pine abundances throughout the area ranging from zero to 150 colonists per ha (0–30 per 200 m^2^ area, Fig. 4A). According to the regional pattern of *P. halepensis* propagule pressure, high propagule pressure is expected only immediately adjacent to seed source stands, while most of the region is exposed to very low propagule inputs (Fig. 4B). However, it is important to remember that additional inputs from local seed sources (mature pines within woodlands) are not included in this map. Although we assume local seed contribution to be negligible in most of the area, this is not true in all cases and may explain site-specific areas of strong pine colonization not predicted in our maps. It seems that much of the heterogeneity in the pattern of *P. halepensis* abundance is related to the highly variable and patchy pattern of the resistance to overall colonization (Fig. 4C) interacting with propagule pressure variability (Fig. 4B). Moreover, local seed sources may further increase the variability of the pattern of pine propagule pressure.

## Discussion

We used inverse modeling to unravel the large-scale and long-term patterns of a complex process of species colonization in heterogeneous human-modified landscapes. Our models provided measures of the factors that control propagule pressure at different spatial scales by directly analyzing successful establishment. Furthermore, our analysis provided quantitative assessment of the factors that determine habitat resistance to colonization, suggesting underlying mechanisms of competition and facilitation. The regional scope of our study allowed the simultaneous assessment of the relative importance of a variety of processes that affect overall colonization acting at a wide range of spatial scales. Although the models explain a relatively low percentage of the regional variation in the density of pine colonization, we consider the results to be robust given the wide range of each of the biotic and abiotic factors included in the study, and the enormous heterogeneity in this landscape. The residual variation may reflect fine scale dynamics due to local processes such as predation pressure, soil moisture conditions, and disturbance.

### Factors that control *P. halepensis* colonization

Our results demonstrate the existence of a “background” regional propagule pressure that is independent of the abundance and proximity of nearby pine forests. In effect, virtually all of the woodlands in the Mediterranean region of Israel experience some level of *P. halepensis* propagule pressure, presumably as a result of the combination of the long tail of the dispersal kernel of wind-dispersed *P. halepensis* seeds [24], [41], the widespread distribution of pine forests in the area (Fig. 1) [33], [51] and sporadic long-distance dispersal events [52]. The overall propagule pressure is significantly enhanced by the presence of pine forests within the immediate surrounding landscape, but our results suggest that the actual configuration of seed sources is only quantitatively important for pine stands within 200 m of woodlands (Fig. 3A). The input of these wind dispersed seeds appears to be associated with eastern and north-western winds, which correspond to the autumn “Sharav” warm easterly winds and to the north-western winds that prevail on cold autumn and winter days respectively [53]. The patterns we found for colonization pressure by the wind-dispersed seed of *P. halepensis* differ dramatically from the distance-independent and density-dependent inputs of the large bird-dispersed acorns of *Q. caliprinos* in the reciprocal process of colonization of planted pine forests by oaks [7].

While we found little evidence for the importance of wind directions to patterns of seed dispersal, wind direction may turn out to play a much stronger role under specific site conditions. The relatively minor role of local propagule pressure – i.e. the small contribution of seeds from mature established pines within the woodland sites – in part reflects the current low densities of reproductive pines in the sites. Thus, while the emergence of local seed sources could serve to accelerate the colonization process in the future, at present this is playing only a minor role in the region.

Our results suggest that the potential colonization of natural ecosystems by the planted pine will be a very heterogeneous process, strongly controlled by local, site-specific factors (Fig. 4; e.g., [54]). The combined effects of strong resistance to pine colonization in sites with low woody vegetation cover (which is equivalent in these ecosystems to high herbaceous cover), and improved colonization under grazing suggest that pine colonization is constrained by strong competition with herbaceous vegetation [55], [56], [57]. At the extremes of the range of these factors, reduced potential establishment in high precipitation and high woody cover indicates that pine colonization may also be slightly limited by competition with the local woody community. This is presumably related to the shade intolerance of pines [58]–[61]. This is in contrast with the process of colonization of planted pine forests by oaks, where colonization is not sensitive to precipitation and is improved in the mid-range of biotic conditions (pine forest age and density and grazing) [7].

### Patterns of *P. halepensis* colonization

It is worth noting that pines are successfully colonizing a wider range of physical site conditions (bedrock, soils, and vegetation) than have been traditionally considered suitable pine habitats in the region [62]. Overall, the patterns of potential colonization indicate that pine colonization under the current conditions is similar to but not entirely predictable by previous knowledge of pine ecology. Naturally occurring *P. halepensis* trees in Israel have been considered as dominants only on Rendzina soils developed on marl and chalk bedrock [63], [64], although in the broader Mediterranean Basin the species also occurs in sites with limestone and dolomite bedrock [65]. Furthermore, in floristic analyses *P. halepensis* and oak-maquis have been described as distinct ecosystems occupying unique habitat conditions, and pine-oak forests have been described only under specific conditions [32]. Our results show that pine colonization patterns reflect these differences in the favorability of different combinations of bedrock and soil type, but also allow colonization of what was formerly considered pure oak habitats. Furthermore, although *P. halepensis* is highly drought resistant [42], our model predicts that the densities of pine colonists will be higher in mesic sites but not at the extremes of the range of precipitation in our sites [43]. These findings suggest that the future distribution of pines may not be predictable from the site requirements that have been typically found for pines in landscapes that are less impacted by afforestation and its consequences on propagule pressure.

The integration of propagule pressure and habitat resistance provides a more complete picture of the factors that lead to very heterogeneous colonization across the study region. Although we found a constant background propagule arrival throughout the region, the effective densities of colonists are dramatically reduced by the impacts of local resistance, leading to zero colonization in a quarter of the sites (e.g. in sites with low woody cover and strong competition from herbaceous vegetation). For example, the maps of expected abundance show that the densities of pine colonists in large areas that only receive the regional propagule input would range from zero to a maximum of 100 pines ha^−1^. Very high densities of colonists, on the other hand, occur where the total propagule pressure is more than six times larger than the regional background pressure (Fig. 4B) combined with high colonization potential. Our findings indicate that while both seed and site availability are important for understanding colonization patterns, the factors that control site limitation will eventually determine presence/absence of the colonist [66].

### A general framework for analyzing species colonization

The strength of our approach lies in its ability to provide quantitative explanations that integrate process that occurred over a large temporal scale and over a wide spatial extent. For instance, the propagule pressures estimated in our analysis represent the cumulative seed inputs over the entire time in which seeds of *P. halepensis* have been available in the region. The age distribution of the colonists gives further insight into temporal patterns within this time frame. The decline in frequency of older pine colonists and the lack of colonists older than 45 years suggest an acceleration of pine colonization 20–30 years ago. This may reflect seed production from maturing *P. halepensis* forests planted widely in the 1950–1970s [51], [67]. The age structure also suggests lower rates of colonization in the past decade. This may be related to variation in precipitation, as this period has been characterized by below-average rainfall in the region, and high rates of pine recruitment occur during years with above-average precipitation [34]. In terms of spatial heterogeneity, our analysis provides measures of the differences in both seed pressures and establishment potentials throughout the large study region, which imply that colonization will form a continuum of pine abundance. Thus, at the large extent of this analysis colonization is not a uniform process which suggest that this colonization is not expected to lead to the simplification of the host ecosystem [68] or homogenization of the landscape [69].

Our analyses provide a basis for at least first-order projections of how future distributions and densities of colonizing pines in these woodlands will vary as a function of changes in: (*i*) the distribution and abundance of pine seed sources, and (*ii*) local resistance factors – particularly precipitation as a result of climate change [70], and grazing regimes, as a result of socio-economic processes. A more thorough assessment of future rates of colonization by pines will need to consider the ways in which changes in the abundance of pines alter the structure and function of the woodland ecosystems (e.g., [10], [12], [14], [71]).

### Management implications

Our findings have a variety of management implications. We found that the densities of *P. halepensis* colonists, when present, are close to typical stand densities, indicating that without significant thinning in the future this could result in a mixture of maquis vegetation and pine trees of an intermediate to high density. Formation of such dense woody ecosystems will have important implications for fire hazards and fire management [72]. The large scale of our analysis provides important insights for management. For example, our analysis shows that approximately 29% of the area of shrublands and woodlands in our study region occurs within 200 m of a pine seed source. Thus, almost a third of the Mediterranean woodlands of Israel are exposed to strong pine propagule pressure. But this propagule pressure comes from a relatively small fraction (22%) of the pine forests in the study area (i.e. stands within 200 m distance to woodlands). Within areas exposed to strong propagule pressure, some habitat conditions allow the highest colonization (e.g., chalk and marl bedrock, intermediate precipitation, moderate levels of woody vegetation cover, and intermediate to heavy grazing) and should therefore receive special management attention.

## Conclusions

Many recent ecological issues, especially those that deal with changes in species distributions or community composition as a result of human action, are at their core related to colonization processes. Our study addresses the complex process of colonization of heterogeneous landscapes by *P. halepensis* with a set of simple rules that control the process. We show how variation in colonization rates– due to landscape-scale heterogeneity in both colonization pressure and resistance to colonization – can be expected to produce a diversity of new ecosystems. Analysis of the colonization process gave insights to the spatial dynamics of pine recruitment, enabled the projection of expected distributions, and provided guidelines for decision-making and management. Future implementations of the inverse modeling approach will provide new perspectives for the study and management of species recruitment and invasion.

## Supporting Information

Figure S1 **Age distribution of all** ***Pinus halepensis*** **colonists in woodlands and shrublands of the Mediterranean region of Israel (** ***n*** **= 601).** The number of whorls is used as a surrogate for pine age. The distribution of <5 year old pines is partial since the survey included only pines >50 cm tall.
(TIF)

Table S1 **Correlation matrix for all the habitat resistance factors.**
(DOC)
