# Supplementary figures and images for: An Integrative Analysis of the Dynamics of Landscape- and Local-Scale Colonization of Mediterranean Woodlands by Pinus halepensis
Source: PLoS One. 2014 Feb 28;9(2):e90178. doi: 10.1371/journal.pone.0090178 (PMC3938658; doi:10.1371/journal.pone.0090178)

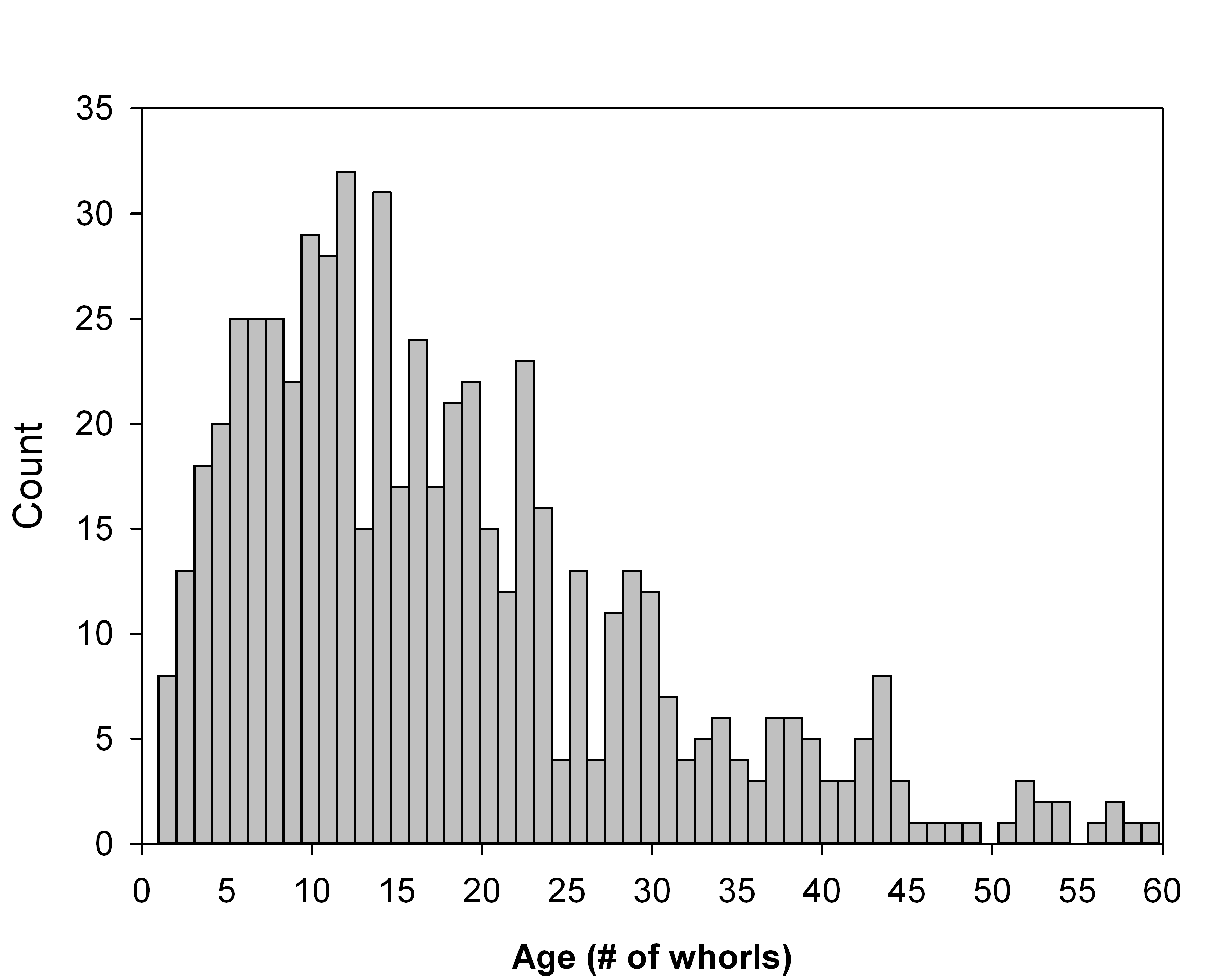

Supplement: Figure S1 — Age distribution of all Pinus halepensis colonists in woodlands and shrublands of the Mediterranean region of Israel ( n = 601). The number of whorls is used as a surrogate for pine age. The distribution of <5 year old pines is partial since the survey included only pines >50 cm tall. (TIF) [file pone.0090178.s001.tif]
