# Supplementary material for: An Integrative Analysis of the Dynamics of Landscape- and Local-Scale Colonization of Mediterranean Woodlands by Pinus halepensis
Source: PLoS One. 2014 Feb 28;9(2):e90178. doi: 10.1371/journal.pone.0090178 (PMC3938658; doi:10.1371/journal.pone.0090178)
Supplement: Table S1 — Correlation matrix for all the habitat resistance factors. (DOC) [file pone.0090178.s002.doc]

**Table S1.** Correlation matrix for all the habitat resistance factors. Pearson's product-moment correlation are significant only between precipitation and woody vegetation cover (t = 7.5112, df = 468, *p* < 0.001) and between precipitation and soil type (t = -8.0815, df = 468, *p* < 0.001).

|  | SOIL | GRAZE | PRECIPITATION | WOODY | ROCK |
| --- | --- | --- | --- | --- | --- |
| SOIL | 1 | 0.0306 | **-0.34995** | -0.0278 | 0.036946 |
| GRAZE |  | 1 | 0.082346 | -0.04284 | -0.05675 |
| PRECIPITATION |  |  | 1 | **0.327998** | -0.03818 |
| WOODY |  |  |  | 1 | -0.04167 |
